# Antihypertensive medications are associated with the risk of kidney and bladder cancer: a systematic review and meta-analysis

**DOI:** 10.18632/aging.102699

**Published:** 2020-01-22

**Authors:** Yuxiu Xie, Peng Xu, Meng Wang, Yi Zheng, Tian Tian, Si Yang, Yujiao Deng, Ying Wu, Zhen Zhai, Qian Hao, Dingli Song, Dai Zhang, Zhijun Dai

**Affiliations:** 1 Department of Breast Surgery, The First Affiliated Hospital, College of Medicine, Zhejiang University, Hangzhou, China; 2 Cancer Center, Union Hospital, Tongji Medical College, Huazhong University of Science and Technology, Wuhan, China; 3 Department of Oncology, The Second Affiliated Hospital of Xi’an Jiaotong University, Xi’an, China

**Keywords:** antihypertensive medications, kidney cancer, bladder cancer, meta-analysis, risk

## Abstract

Several studies have indicated that the use of antihypertensive medications may influence the incidence of bladder/kidney cancer, with some scholars refuting any such association. Hence, a systematic review is needed to verify this linkage. we comprehensively searched PubMed, Embase, Web of Science, and the Cochrane Library for original studies reporting a relationship between antihypertensive medications and risk of bladder/kidney cancer. We included 31 articles comprising 3,352,264 participants. We found a significant association between the risk of kidney cancer and any antihypertensive medications use (relative risk (RR) = 1.45, 95% CI 1.20-1.75), as well as angiotensin-converting enzyme inhibitors (RR = 1.24, 95% CI 1.04-1.48), angiotensin II receptor blockers (ARB) (RR = 1.29, 95% CI:1.22-1.37), beta-blockers (RR = 1.36, 95% CI 1.11-1.66), calcium-channel blockers (RR = 1.65, 95% CI 1.54-1.78) and diuretics (RR = 1.34, 95% CI 1.19-1.51). In case of bladder cancer, a statistical significance was observed with the use of ARB (RR = 1.07, 95% CI 1.03-1.11) but not with the other antihypertensive medications. There was a linear association between the duration of antihypertensive medications and the risk of kidney cancer (P = 0.061 for a non-linear trend) and the pooled RR for the per year increase in antihypertensive medications duration of use was 1.02 (95% CI: 1.01-1.02). Our results indicate that there is a significant association between each class of antihypertensive medications and the risk of kidney cancer, and this trend presented as a positive linear association. Furthermore, the use of ARB has been linked to the risk of bladder cancer.

## INTRODUCTION

Hypertension is a highly prevalent chronic disease worldwide in the elderly, necessitating the long-term use of various antihypertensive medications to prevent cardiovascular morbidity and mortality. However, several studies have demonstrated the potential risks of antihypertensive medications including orthostatic hypotension, falls, cognitive decline, dementia, fractures, diabetes, and cancer [1].

Preclinical experimental studies have indicated that antihypertensive medications, such as angiotensin-converting enzyme inhibitors (ACEI), angiotensin II receptor blockers (ARB), calcium-channel blockers (CCB) and beta-blockers (BB), can facilitate or interfere with tumor cell proliferation, migration, and apoptosis, as well as angiogenesis [2–4]. For example, it was observed that angiotensin II type I receptor (Ang II AT1R) was highly expressed in bladder cancer cells of high-stage and/or high-grade tumors and Ang II AT1R signaling could induce the expression of the vascular endothelial growth factor (VEGF) [5]. Moreover, ACEIs and ARBs have demonstrated anti-angiogenetic effects, reducing VEGF expression in bladder malignancies [4]. Notably, telmisartan played a potent anti-proliferative role in urological cancer cells through the peroxisome proliferator-activated receptor gamma (PPAR-γ) [6]. However, the thiazide diuretic treatment in rats could result in degenerative changes including cell apoptosis and tumor cell markers in the distal tubule [7].

A parallel, randomized, double-blind, controlled, clinical trial assessing the effects of candesartan observed an unexpected phenomenon. Reportedly, the candesartan group (3803 patients) displayed a higher cancer mortality than patients treated with the placebo (3796 patients) (2.3% vs 1.6%, *p*=0.038) [8]. Furthermore, several meta-analyses demonstrated that antihypertensive medications usage may influence the incidence of cancer [9–13]. In 2010, a meta-analysis based on nine randomized trials demonstrated that, compared to the placebo or comparator agents, ARB therapy indicated a modestly increased risk of new cancer occurrence, with a significant association observed in lung cancer, but not prostate and breast cancer, among the solid organ cancers examined [9]. However, a network meta-analysis refuted the relative risk increase between cancer or cancer-related death and the use of most antihypertensive medications classes [10].

Notably, the morbidity and mortality of kidney and bladder cancers are increasing, and the prognosis remains unfavorable. According to the 2018 *Global cancer statistics*, it was estimated that 549,393 individuals were newly diagnosed with bladder cancer, with 199,922 patients’ deaths reported from the disease. Additionally, kidney cancer reportedly accounted for approximately 2.2% of all new cancer cases and 175,018 deaths worldwide in 2018 [14]. Therefore, there is an urgent need to elucidate relevant mechanisms and risk factors. In previous epidemiologic studies, several potential risk factors for bladder/kidney cancer have been investigated including age, obesity, cigarette smoking, a family history of bladder or kidney cancer, exposure to certain chemicals, and sex, with men indicating a higher incidence of both cancers compared to women [15, 16]. However, with the emergence of observational data, the association between antihypertensive medications and the risk of kidney/bladder cancer is more controversial. Therefore, we conducted this review to evaluate the existence of an association between these factors.

## RESULTS

### Characteristics and quality of studies

**Figure 1:**
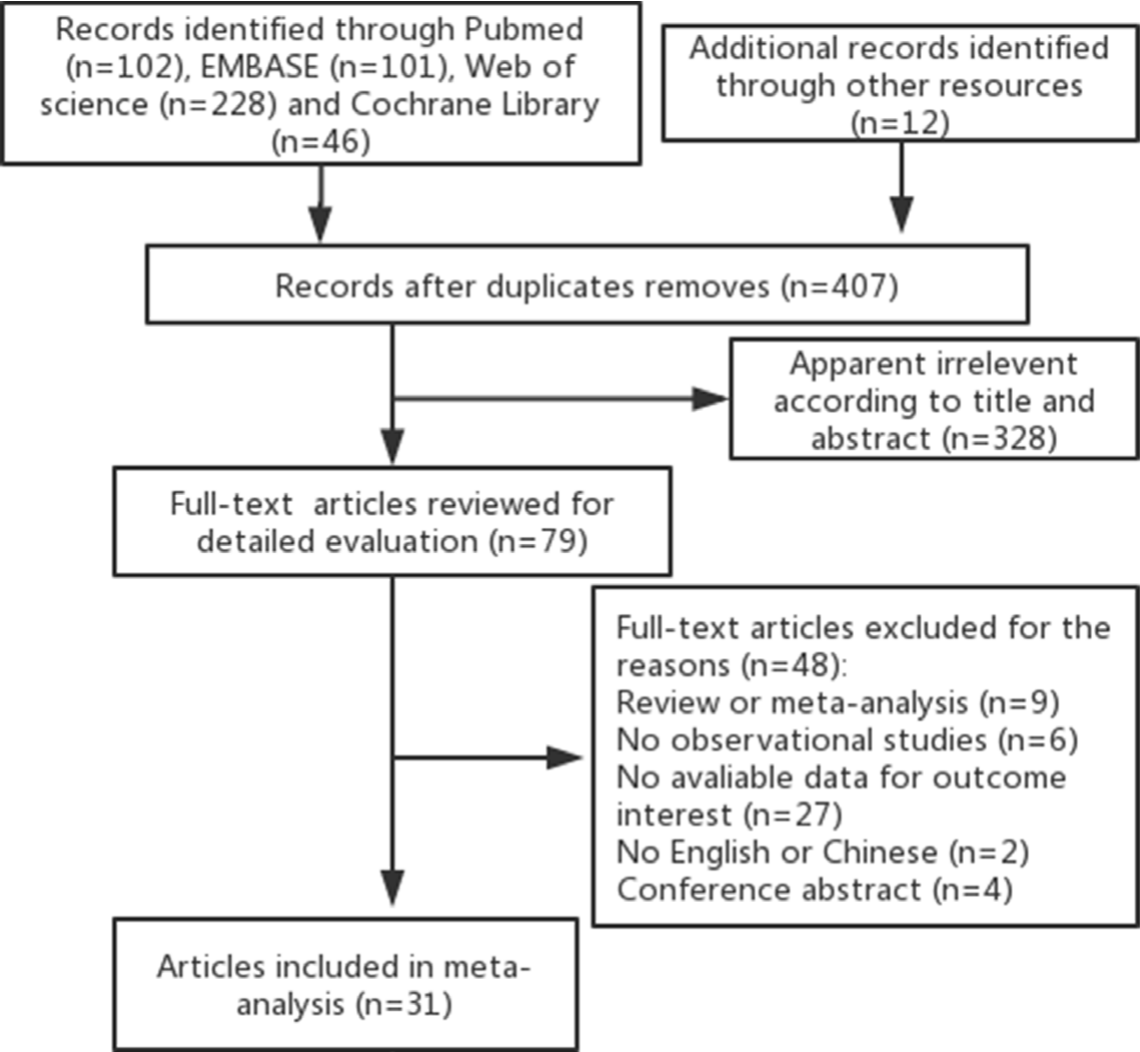
**Flow chart of selection process of observational studies.**

As illustrated in Figure 1, our initial search identified 407 potentially relevant citations from online databases and reference lists, from which 328 were excluded after screening the titles and abstracts. Ultimately, 31 articles met the inclusion criteria and were finally included in this meta-analysis [17–47]. This selection consisted of 18 articles designed as case-control studies and 13 articles designed as cohort studies, with approximately 3,352,264 participants. Moreover, these articles were regarded as independent studies since the role of antihypertensive medications in the risk of bladder/kidney cancer was assessed according to the different antihypertensive medications classes (ACEI, ARB, BB, CCB, diuretics or any antihypertensive medications), cancer sites (kidney, bladder), and gender. The characteristics of the articles are listed in Table 1. Data collection for antihypertensive medications use was not consistent across studies, with most using questionnaires or prescription database reviews. Cancer cases were ascertained by cancer registries or medical records from the included studies. Most studies controlled potential confounding factors (age, gender, BMI, smoking, hypertension) by matching or adjustments; however, inconsistencies among the adjustments in each study were observed. The scores of the Newcastle-Ottawa Scale (NOS) quality assessment ranged from 6 to 8 and are listed in Table 1.

**Table 1:** Characteristics of the articles included in the meta-analysis.

**Author, yr [Ref]**	**Location**	**Study period**	**Age (yr)**	**No.of cases / participants**	**Class of medication (reference group)**	**Outcome**	**Type of study**	**Adjustment for covariates**	**QS**
Assimes TL 2008	Canada	1980-2003	71.8	11,697/77,887	BB, CCB, RASIs (thiazide diuretics)	risk of kidney cancer	case-control	age, all measured comorbid conditions, and exposure to all other classes of antihypertensive not of interest	7
Chow WH 1995	USA	1988-1990	64(20–79)	151/842	Diuretics, AHT (no use)	risk of renal cell cancer	case-control	age, sex, smoking, BMI, hypertension	8
Chuang YW 2017	China	2005-2011	71	32,167/64,334	ACEI, ARB, CCB, Diuretics (no use)	risk of bladder cancer and kidney cancer	case-control	age, sex, diabetes, COPD, stroke, coronary arterial disease, related comorbidities such as benign prostate hyperplasia, CKD, urinary stones, and urinary tract infection	7
Colt JS 2017	USA	2002-2007	20–79	1,217/2,452	ACEI, CCB, Diuretics (no use)	risk of renal cell cancer	case-control	age, race, sex, region, education, smoking status, body mass index, family history of cancer, and self-reported extent of hypertension control	7
Finkle WD 1993	USA	1980-1989	59.6	191/382	Diuretics (no use)	risk of renal cell cancer	case-control	hypertension, smoking, obesity	6
Guercio V 2019	Italy	2003-2014	66.5	690/1355	CCB (no use)	risk of bladder cancer	case-control	age, sex, study centre, year of interview, education, tobacco smoking and diabetes	7
Hiatt RA 1994	USA	1964-1989	50.7	257/514	Diuretics (no use)	risk of renal cell cancer	case-control	smoking, BMI, hypertension, history of kidney infection	7
Hole DJ 1998	UK	1980-1995	52	2,297/5,207	CCB (no use)	risk of bladder cancer and kidney cancer	case-control	age, sex, year of observation, smoking habit	7
Jiang X 2010	USA	1987-1996	25–64	1,585/3,170	Diuretics, AHT (no use)	risk of bladder cancer	case-control	smoking, education, lifetime use of ‘non-steroidal anti-inflammatory drugs (NSAIDs), intake of carotenoids (quintiles), ever held a high-risk occupation	7
Kreiger N 1993	Canada	1986-	25–69	518/1,899	Diuretics (no use)	risk of renal cell cancer	case-control	age, smoking, hypertension, combined Quetelet index	7
McCrediM 1992	Australia	1989-1991	20–79	636/1,159	AHT, Diuretics, BB (no use)	risk of renal cell cancer	case-control	age, sex, smoking, obesity, hypertension, terms for diuretics and potassium supplements, method of interview	6
McLaughli JK 1995	Australia	1989-1991	20–79	1,732/4,041	Diuretics, AHT (no use)	risk of renal cell cancer	case-control	age. sex, BMI, smoking, hypertension, center	8
Mellemgaard A 1994	Denmark	1989-1992	20–79	368/764	AHT, ACEI, BB, CCB, Diuretics (no use)	risk of renal cell cance	case-control	age, smoking, socioeconomic status, BMI, hypertension	8
RosenberL 1998	USA	1983-1996	40–69	9,513/16,005	ACEI, BB, CCB (no use)	risk of kidney and bladder cancer	case-control	age, physician visits 2 years previously	7
Shapiro JA 1999	USA	1980-1995	18–84	238/854	ACEI, BB, CCB, Diuretics (no use)	risk of renal cell cancer	case-control	age, BMI	7
Weinman S 1994	USA	1960-1991	63(W)/ 64(M)	206/498	AHT, BB, Diuretics (no use)	risk of renal cell cancer	case-control	age, sex, date of entry into the Health plan, number of months in the Health plan	7
Yu MC 1986	USA	1975-1979	46.1	160/320	Diuretics (no use)	risk of renal cell cancer	case-control	sex, birth date (within 5 yr), race, and neighborhood of residence at time of diagnosis.	7
Yuan JM 1998	USA	1986-1994	58.8	1,204/2,408	Diuretics (no use)	risk of renal cell cancer	case-control	education, BMI, hypertension	8
Braun S 1998	Israel	1990-1993	59.8	43/11,575	CCB (no use)	risk of bladder and kidney cancer	cohort	age, sex, smoking	6
Chang PY 2015	China	2000-2011	54.6	70/24,238	BB (no use)	risk of bladder and kidney cancer	cohort	age, sex, CCI score, hypertension, angina pectoris, aroxysmal supraventricular tachycardia, hypertensive renal disease, essential tremor, anxiety, thyrotoxicosis, migraine and medication of statins, metformin, aspirin, a-blockers, other b-blockers.	8
Flaherty KT 2005	USA	1976-2000	42.4(W)/ 54 (M)	265/167,144	Diuretics (no use)	risk of renal cell cancer	cohort	age, hypertension, BMI	7
Fraser GE 1990	USA	1977-1982	72.3	14/34,198	AHT (no use)	risk of renal cancer	cohort	age, sex	7
Fryzek JP 2005	Denmark	1989-2002	62(30–85)	330/113,298	ACEI, ARB, BB, CCB, Diuretics (no use, BB)	risk of renal cell cancer	cohort	age, sex, calendar period	7
Heath CW 1997	USA	1982-1989	>=30	335/998,904	Diuretics, AHT (no use)	risk of renal cell cancer	cohort	age, race, educabon, smoking, BMI, acetaminophen use, history of urologic disease, and asbestos exposure.	7
MackenzieTA 2016	USA	2006-2012	75.1(P)/ 76.7(I)	4433/1,161,443 (P), 320,090(I)	ACEI, ARB (no use)	risk of bladder cancer	cohort	age, gender, race, low-income subsidy, alcohol, chronic obstructive lung disease and/or tobacco use, obesity, diabetes complications and Charlson comorbidities	8
Prineas RJ 1997	USA	1986-1993	55–69	62/35,192	Diuretics (no use)	risk of renal cell cancer	cohort	age, maximum weight, WHR, uncertainty about blood transfusion history	7
Schouten LJ 2005	Netherlands	1986-1997	61.9	337/4,774	AHT, BB, Diuretics (no use)	risk of renal cell cancer	cohort	age, sex, BMI, smoking	7
Setiawan VW 2007	USA	1993-2002	59	347/161,126	Diuretics (no use)	risk of renal cell cancer	cohort	BMI, smoking (status and pack-years of smoking), alcohol drinking, hypertension, and physical activity.	8
Sugiura R 2012	Japan	2001-2004	65	1,024/2,049	ARB (non ARB standarzed AHT)	risk of bladder and kidney cancer	cohort	age, sex, co-morbidities, pharmacotherapy.	7
Tseng CH 2011	China	2003-2005	NR	589/998,947	ACEI, Diuretics (no use, BB)	risk of bladder cancer	cohort	NR	7
Weikert S 2008	Europe	1992-2004	25–70	250/296,638	AHT (no use)	risk of renal cell cancer	cohort	sex, body mass index, education, duration of smoking, smoking status, systolic blood pressure	8

QS: NOS quality assessment; AHT: antihypertensive medications; ACEI: angiotensin-converting enzyme inhibitors; ARB: angiotensin II receptor blockers; CCB: calcium-channel blockers; BB: beta-blockers; RASIs: renin angiotensin system inhibitors; BMI: body mass index; M:men, W: women; P; Prevalent cohort; I: Incident cohor.

### Antihypertensive medications and the risk of bladder cancer

**Figure 2:**
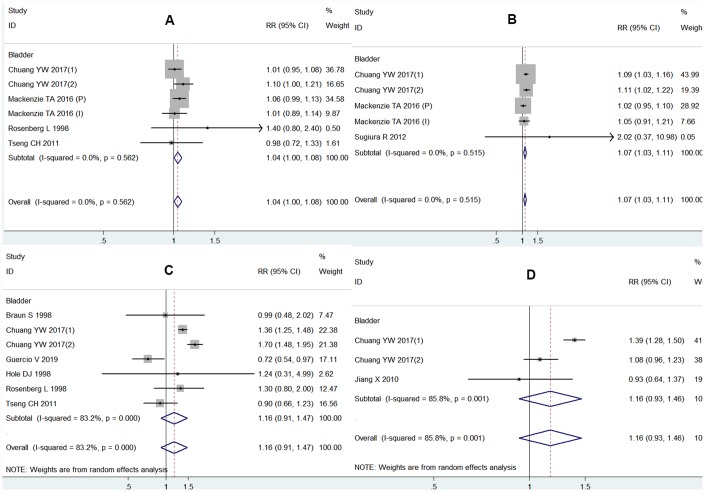
**Forest plot of association between using each class of antihypertensive medications and bladder cancer risk:** (**A**) ACEI and bladder cancer risk; (**B**) ARB and bladder cancer risk; (**C**) CCB and bladder cancer risk; (**D**) diuretics and bladder cancer risk.

As shown in Figure 2, the outcomes based on five studies indicated that ARB use was associated with an increased risk of bladder cancer (relative risk (RR) = 1.07, 95% Confidence Interval (CI) 1.03-1.11) with little heterogeneity (I^2^ = 0.0%), with no statistical significance demonstrated for other antihypertensive medications usage. Two studies adjusted for hypertension recorded that a significant association existed with ARB therapy and the risk of bladder cancer (RR = 1.10, 95% CI 1.04-1.15). However, two studies adjusted for smoking demonstrated no relevant association between ARB therapy and cancer risks (RR = 1.03, 95% CI 0.96-1.10). Moreover, after adjusting for hypertension, the results of CCB or diuretic therapy shifted from no statistical significance to statistically significant for bladder cancer (Table 2).

**Table 2:** The results of the association between the each class of antihypertensive medications and bladder cancer risk.

**Comparison**	**ACEI vs nonuse**				**ARB vs nonuse**			
**Category**	**n**	**RR 95% CI**	**I^2^(%)**	***P(h)***	**n**	**RR 95% CI**	**I^2^(%)**	***P(h)***
Bladder	6	1.04 (1.00,1.08)	0.0	0.562	5	1.07 (1.03, 1.11)	0.0	0.515
**Adjustment of individual estimates for hypertension**								
Yes	2	1.04 (0.98, 1.09)	52.9	0.145	2	1.10 (1.04, 1.15)	0.0	0.740
No	4	1.05 (0.99, 1.11)	0.0	0.636	3	1.03 (0.96, 1.10)	0.0	0.691
**Adjustment of individual estimates for smoking**								
Yes	2	1.05 (0.99, 1.11)	0.0	0.5	2	1.03 (0.96, 1.10)	0.0	0.723
No	4	1.04 (0.99, 1.09)	11.7	0.334	3	1.10 (1.04, 1.15)	0.0	0.737
**Comparison**	**CCB vs nonuse**				**Diuretics vs nonuse**			
**Category**	**n**	**RR 95% CI**	**I^2^(%)**	***P(h)***	**n**	**RR 95% CI**	**I^2^(%)**	***P(h)***
Bladder	7	1.16 (0.91, 1.47)	83.2	0.000	3	1.16 (0.93, 1.46)	85.8	0.001
**Adjustment of individual estimates for hypertension**								
Yes	2	1.51 (1.21, 1.88)	86.3	0.007	2	1.23 (1.96, 1.58)	91.2	0.001
No	5	0.90 (0.73, 1.13)	19.1	0.293	1	0.93 (0.64, 1.36)	-	-
**Adjustment of individual estimates for smoking**								
Yes	3	0.77 (0.59, 1.00)	0.0	0.570	1	0.93 (0.64, 1.36)	-	-
No	4	1.33 (1.07, 1.65)	81.1	0.001	2	1.23 (1.96, 1.58)	91.2	0.001

ACEI: angiotensin-converting enzyme inhibitors; ARB: angiotensin II receptor blockers; CCB: calcium-channel blockers; BB: beta-blockers; *(h):* heterogeneity; n: number of study.

### Antihypertensive medications and kidney cancer risk

**Figure 3:**
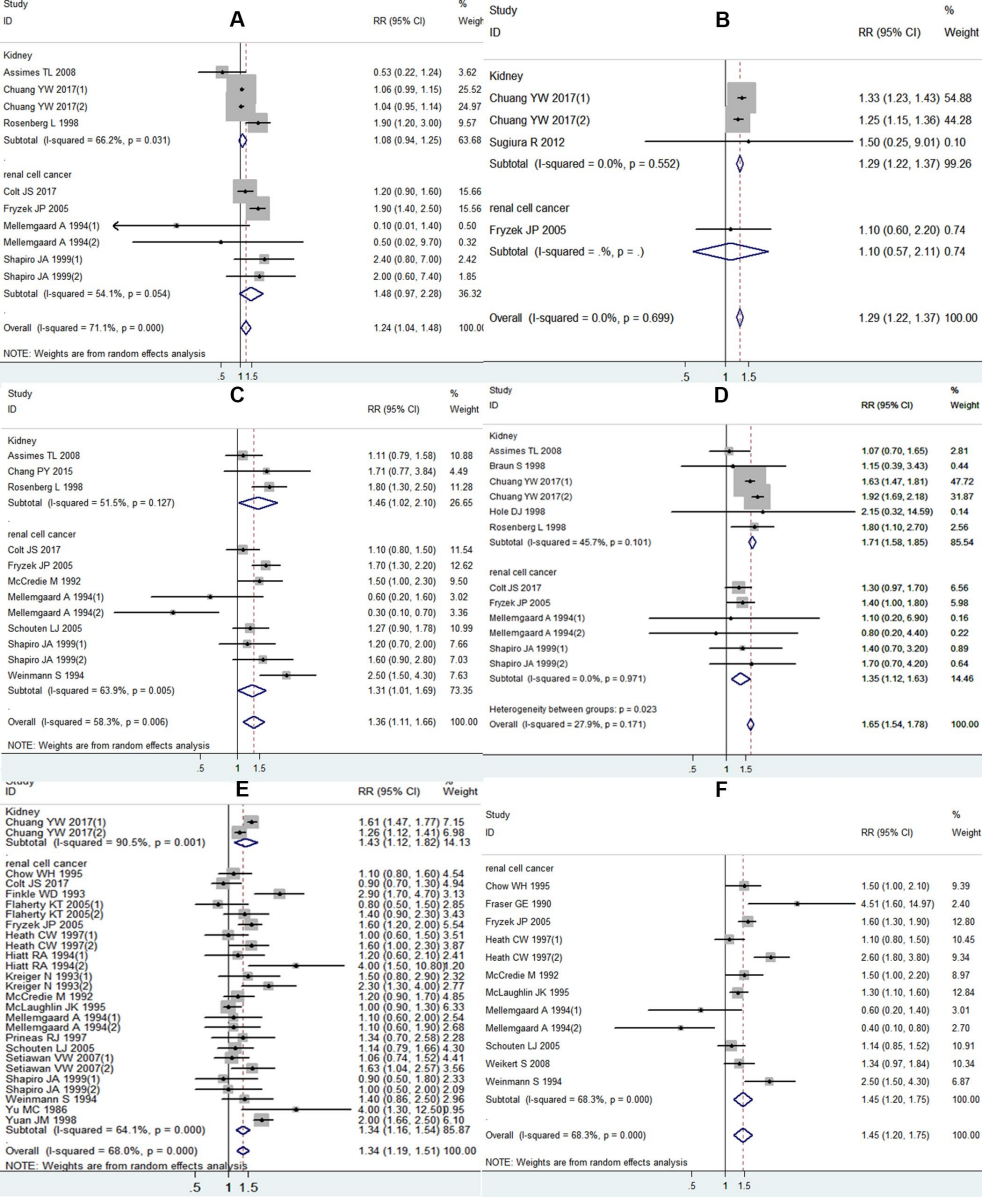
**Forest plot of association between using each class of antihypertensive medications and kidney cancer risk:** (**A**) ACEI and kidney cancer risk; (**B**) ARB and kidney cancer risk; (**C**) BB and kidney cancer risk; (**D**) CCB and kidney cancer risk; (**E**) diuretics and kidney cancer risk; (**F**) any antihypertensive medications and kidney cancer risk.

As shown in Figure 3A, ten studies reported the association between ACEI and the risk of kidney cancer. We observed a significant overall effect size estimate for ACEI therapy and the risk of kidney cancer in the pooled RR (RR = 1.24, 95% CI 1.04-1.48). An obvious heterogeneity existed among the pooled RR studies (I^2^ = 71.1%). No association between the risk of kidney cancer and ACEI use was observed upon evaluating the studies grouped according to gender. Moreover, the statistical significance disappeared after adjusting for hypertension or smoking (Table 3).

**Table 3:** The results of the association between the each class of antihypertensive medications and kidney cancer risk.

**Comparison**	**ACEI vs nonuse**				**ARB vs nonuse**				**BB vs nonuse**			
**Category**	**n**	**RR 95% CI**	**I^2^(%)**	***P(h)***	**n**	**RR 95% CI**	**I^2^(%)**	***P(h)***	**n**	**RR 95% CI**	**I^2^(%)**	***P(h)***
Kidney	4	1.08 (0.94, 1.25)	66.2	0.031	3	1.29 (1.22, 1.37)	0.0	0.552	3	1.46 (1.02, 2.10)	51.5	0.127
Renal cell	6	1.48 (0.97, 2.28)	54.1	0.054	1	1.10 (0.57, 2.11)	-	-	9	1.31 (1.01, 1.69)	63.9	0.005
All kidney	10	1.24 (1.04, 1.48)	71.1	0.000	4	1.29 (1.22, 1.37)	0.0	0.699	12	1.36 (1.11, 1.66)	58.3	0.006
**Gender**												
Women	3	1.04 (0.95, 1.14)	0.0	0.534	1	1.25 (1.15, 1.36)	-	-	2	0.73 (0.14, 3.74)	88.2	0.004
Men	3	1.03 (0.38, 2.80)	64.8	0.058	1	1.33 (1.23, 1.43)	-	-	2	0.99 (0.54, 1.82)	26.5	0.243
All	4	1.39 (0.93, 2.07)	73.8	0.010	2	1.14 (0.62, 2.10)	0.0	0.750	8	1.48 (1.23, 1.77)	46.9	0.068
**Adjustment of individual estimates for hypertension**												
Yes	5	1.06 (0.99, 1.13)	13.0	0.331	2	1.29 (1.22, 1.37)	14.0	0.281	5	1.00 (0.63, 1.58)	64.9	0.023
No	5	1.62 (1.07, 2.44)	50.8	0.087	2	1.14 (0.62, 2.10)	0.0	0.750	7	1.52 (1.26, 1.84)	41.1	0.117
**Adjustment of individual estimates for smoking**												
Yes	4	1.10 (0.33, 3.71)	50.2	0.111					4	0.92 (0.53. 1.60)	71.9	0.014
No	6	1.22 (1.03, 1.45)	79.5	0.000	4	1.29 (1.22, 1.37)	0.0	0.699	8	1.49 (1.22, 1.82)	46.7	0.069
**Comparison**	**CCB vs nonuse**				**Diuretics vs nonuse**				**Any antihypertensive medications vs nonuse**			
**Category**	**n**	**RR 95% CI**	**I^2^(%)**	***P(h)***	**n**	**RR 95% CI**	**I^2^(%)**	***P(h)***	**n**	**RR 95% CI**	**I^2^(%)**	***P(h)***
Kidney	6	1.71 (1.58, 1.85)	45.7	0.101	2	1.43 (1.12, 1.82)	90.5	0.001				
Renal cell	6	1.35 (1.12, 1.63)	0.0	0.971	25	1.34 (1.16, 1.54)	64.1	0.000	12	1.45 (1.20, 1.75)	68.3	0.000
All kidney	12	1.65 (1.54, 1.78)	27.9	0.171	27	1.34 (1.19, 1.51)	68.0	0.000				
**Gender**												
Women	3	1.90 (1.68, 2.16)	0.0	0.525	10	1.58 (1.27, 1.97)	56.4	0.014	2	1.09 (0.17, 6.79)	90.9	0.001
Men	3	1.62 (1.46, 1.80)	0.0	0.845	8	1.16 (0.92, 1.48)	60.6	0.013	2	0.97 (0.61, 1.57)	25.9	0.245
All	6	1.35 (1.15, 1.60)	0.0	0.678	9	1.31 (1.04, 1.64)	79.1	0.000	8	1.48 (1.26, 1.73)	47.4	0.065
**Adjustment of individual estimates for hypertension**												
Yes	5	1.70 (1.57, 1.83)	54.5	0.066	20	1.35 (1.17, 1.54)	74.0	0.000	8	1.34 (1.05, 1.70)	68.3	0.002
No	7	1.40 (1.15, 1.70)	0.0	0.782	7	1.34 (1.07, 1.68)	27.3	0.220	4	1.74 (1.19, 2.53)	72.9	0.011
**Adjustment of individual estimates for smoking**												
Yes	4	1.15 (0.55, 2.40)	0.0	0.890	15	1.33 (1.13, 1.58)	55.9	0.004	10	1.36 (1.09, 1.69)	67.7	0.001
No	8	1.66 (1.54, 1.78)	48.9	0.057	12	1.36 (1.16, 1.60)	72.9	0.000	2	1.86 (1.23, 2.82)	59.1	0.118

ACEI: angiotensin-converting enzyme inhibitors; ARB: angiotensin II receptor blockers; CCB: calcium-channel blockers; BB: beta-blockers; *(h):* heterogeneity; n: number of study.

Four studies reported a connection between ARB therapy and the risk of kidney cancer, with a significant result detected (RR = 1.29, 95% CI: 1.22-1.37) without heterogeneity (I^2^ = 0.0%), as shown in Figure 3B. For studies adjusted for hypertension, the pooled RR was significant (Table 3).

As illustrated in Figure 3C, 12 studies reported an association between BB use and the risk of kidney cancer. We observed an increased risk of kidney cancer with BB therapy (RR = 1.36, 95% CI 1.11-1.66). However, no association between BB therapy and risk for kidney cancer was observed when the RR was pooled based on an adjustment for hypertension or smoking (Table 3).

A total of 12 studies reported a connection between CCB therapy and the risk of kidney cancer. A significant association between CCB and the risk of kidney cancer was established, according to the pooled RR (RR = 1.65, 95% CI 1.54-1.78), as shown in Figure 3D. A modest heterogeneity existed among these studies (I^2^ = 27.9%). A significant association was observed in the gender subgroup and adjustments for hypertension, but not smoking (Table 3).

Twenty-seven studies evaluated the association between the use of diuretics and the risk of kidney cancer. We detected an increased risk of kidney cancer on comparing the use of diuretics versus nonusers in the pooled RR (RR = 1.34, 95% CI 1.19-1.51), as shown in Figure 3E. Notably, in the subgroup analyses, the association was significant with the adjustment for hypertension and smoking. The pooled RR stratified according to gender demonstrated a significant association for women but not for men (Table 3).

As shown in Figure 3F, 12 studies reported that all antihypertensive medications classes were related to the risk of kidney cancer. As reported, there was an increased risk for kidney cancer (RR = 1.45, 95% CI 1.20-1.75), with some heterogeneity (I^2^ = 68.3%). According to the gender subgroups, antihypertensive medications use in men and women was not associated with the risk of kidney cancer. Regardless of whether the study had been adjusted for hypertension or smoking, a significant relationship between antihypertensive medications use and kidney cancer risk was observed (Table 3).

### Dose-response association between the duration of antihypertensive medications therapy and the risk of kidney cancer

**Figure 4:**
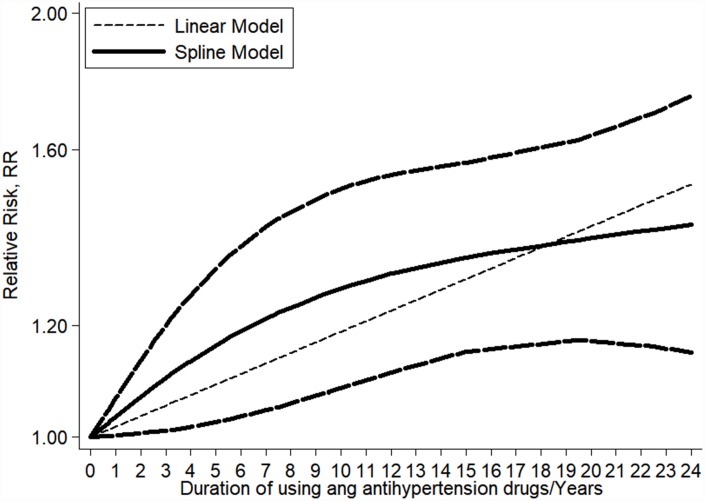
**Linear dose–response meta-analysis between the duration of antihypertensive medications use and kidney cancer risk.**

**Figure 5:**
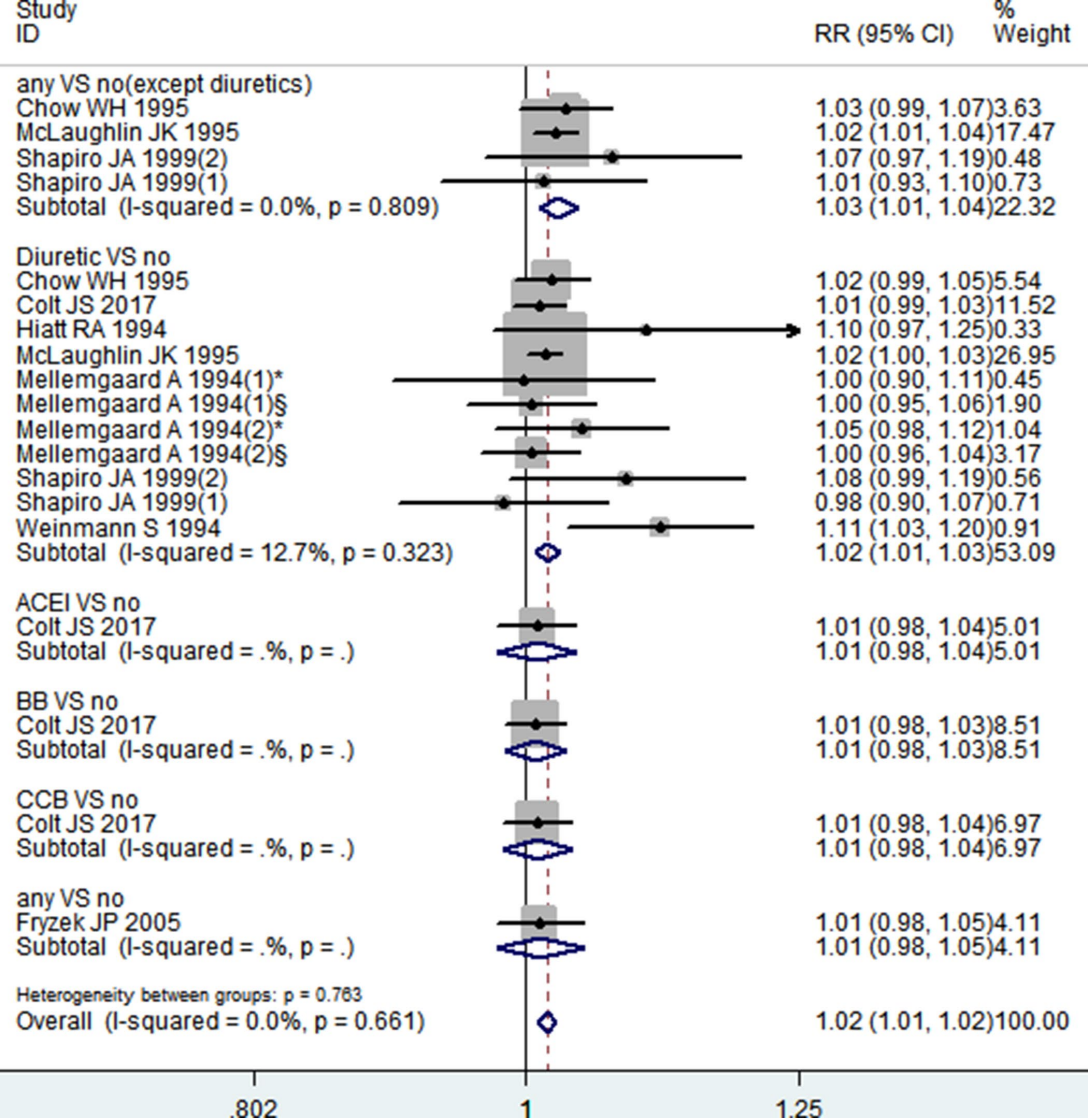
**Forest plot of association between per 1-year increment of using antihypertensive medications and kidney cancer risk.**

We included eight articles in our dose-response analysis [18, 20, 23, 28, 29, 31, 32, 39]. As shown in Figure 4, the results indicated that there was a linear association between the duration of antihypertensive medications therapy and the risk of kidney cancer (*P* = 0.061 for a non-linear trend). The pooled RR for each year of increasing antihypertensive medications use was 1.02 (95% CI: 1.01-1.02), with little heterogeneity among studies (I^2^ = 0.0%, *P* = 0.661) (Figure 5). Particularly, the per year increase in diuretics therapy was associated with a 2% higher incidence of kidney cancer (RR = 1.02, 95% CI: 1.01-1.03). However, a small number of studies researched the relationship between the duration of ACEI, ARB, BB, or CCB use and the risk of kidney cancer, necessitating relevant studies to assess these results.

### Publication bias and sensitivity analysis

Begg’s funnel plot and Egger’s test were performed and found no evidence of publication bias in the analysis, as shown in Supplementary Figures 1 and 2. A sensitivity analysis on the risk of kidney cancer was performed as shown in Supplementary Figure 3. The pooled RR remained statistically significant, indicating that our results are stable. As the number of bladder cancer investigations were limited, a sensitivity analysis was omitted.

## DISCUSSION

The present systematic review with meta-analysis indicates that the risk of bladder cancer is related to ARB, but not with other antihypertensive medications classes. Additionally, we note that ACEI, ARB, BB, CBB, diuretics and all antihypertensive medications classes are associated with a risk of kidney cancer. The results from the dose-response analysis provided evidence that with the prolonged use of antihypertensive medications, the risk of kidney cancer increases.

Notably, the mechanism of association between the risk of bladder/kidney cancer and antihypertensive medications therapy remains unclear. In vitro studies have suggested that ARB increased the risk of cancer by promoting cellular proliferation, angiogenesis, and tumor progression [48]. In contrast, other studies have reported that ACEI and ARB have a possible antitumor effect by reducing angiogenesis in bladder malignancies and renal cell carcinoma [4, 6]. Furthermore, investigators have also reported the antitumor effect of CCB, implicated in the regulation of cell proliferation and calcium influx [49]. In addition, it has been long hypothesized that diuretics have a low-grade carcinogenic effect by targeting the renal tubular cell [43]. For example, it has been reported that rodents developed nephropathy and renal adenomas after diuretic treatment [50]. Preclinical studies have corroborated that various antihypertensive medications classes have effects on cancer cells or in animal models; however, the exact mechanism is unknown. In order to evaluate the existence of a relationship between antihypertensive medications and bladder/kidney cancer risk, we performed this meta-analysis from the clinical point of view.

The observed association between the risk of bladder/ kidney cancer and antihypertensive medications therapy can be explained by factors such as obesity, smoking, alcohol consumption, and hypertension. Hypertension has been documented as a general risk factor for cancer, particularly for renal cell carcinoma [51]. This could be attributed to the common risk factors such as smoking, diabetes, obesity, and alcohol consumption shared between hypertension and cancer. However, the persisting question for decades has been whether the association between antihypertensive medications therapy and the risk of bladder/kidney cancer is independent of hypertension. Several studies support this hypothesis. For example, one study indicated that, even with an adjustment for the use of other antihypertensive medications classes and the time from a hypertension diagnosis to the end of the study, the risk trend for papillary renal cell cancer persisted during the use of diuretics among participants with a history of hypertension [20]. In the subjects with normal blood pressure, diuretic use has also been associated with renal cell carcinoma [21, 33]. However, in studies adjusted for hypertension, no association was observed between antihypertensive medications and the risk of bladder/kidney cancer [28, 29]. Moreover, our result demonstrated a dose-response relationship, reporting an increased risk of kidney cancer risk with the length of exposure to antihypertensive medications. This could indicate that long-time antihypertensive medications therapy is a risk factor for renal cell carcinoma; however, this could simply reflect the increasing risks associated with the duration and severity of hypertension. To resolve this dispute, we conducted subgroup analyses with adjustments for individual estimates of hypertension/smoking. In this meta-analysis, we observed that ARB usage remained a significant risk factor for bladder cancer even after adjusting for hypertension. Notably, the significant association disappeared after adjusting for smoking. In the kidney cancer subgroup, the pooled RRs remain significant with diuretics when adjusted for hypertension and smoking. However, no statistical significance was observed when adjusted for the presence of hypertension or smoking in ACEI, ARB, BB, and CCB therapy. The rationale behind the pooled RRs decreasing or approaching insignificance when adjusted for hypertension or smoking is presumed as follows. First, there are no associations between ARB and bladder cancer risk, and between ACEI or ARB or BB or CCB and the risk of kidney cancer, with significant results attributed to the unadjusted risk factors such as smoking and hypertension. On the other hand, the number of studies adjusted for hypertension or smoking were minimal and the results lacked statistical power. Therefore, it is difficult to ascertain whether ACEI, ARB, BB, and CCB confer an additional risk in bladder/kidney cancer beyond smoking or hypertension.

After adjusting for hypertension and smoking, the RR for diuretics and the risk of kidney cancer was still significant, indicating that diuretics are a risk factor for kidney cancer. Our pooled data also observed that women demonstrate a significant risk of kidney cancer associated with diuretics, but men do not. Several studies have attempted to explain the differential effects of diuretics between sexes. Previously, studies have indicated that women are at a higher diuretic-associated cancer risk than men [42, 44]. The proposed explanation suggested that women were prescribed diuretics more frequently than men, with a higher chemical burden on the tubular cells resulting in carcinogenicity [52]. Additionally, there is a higher estrogen exposure in women, which has been known to enhance the density of the thiazide sensitive NaCl transporter in the distal tubule [53].

This research has several limitations. First, this is a meta-analysis based on observational studies, which are inherently prone to several types of bias including selection bias, detection bias, recall bias, publication bias, and confounding bias. Second, only a small number of studies with data on bladder cancer risk and gender subgroup analysis were included. Consequently, the statistical power in the analyses is insufficient and the results should be interpreted with caution. Third, significant heterogeneity was observed among the studies. Although a sensitivity analysis and subgroup analysis were employed, the heterogeneity persisted.

In conclusion, our meta-analysis suggests that antihypertensive medications therapy, including ACEI, ARB, BB, CCB, and diuretics, is consistently associated with the risk of kidney cancer but not bladder cancer, except for ARB. The longer the duration of antihypertensive medications therapy, the higher the risk of kidney cancer, presenting a positive linear trend. Although our results indicate that the use of antihypertensive medications can slightly increase the risk of kidney cancer, hypertensive patients should continue to stabilize blood pressure with antihypertensive medications to reduce the morbidity and mortality associated with cardiovascular events, while simultaneously undergoing kidney and bladder cancer screening.

## MATERIALS AND METHODS

We followed the Preferred Reporting Items for Systematic reviews and Meta-Analyses (PRISMA) guidance [54].

### Data sources and search strategy

Two investigators independently searched the literature in PubMed, Embase, Web of Science, and Cochrane Library databases from inception to July 2019 using the following text and corresponding range of medication names: “urinary tract cancer or kidney cancer or renal cell cancer or urinary bladder cancer or urethra cancer or kidney carcinoma or renal cell carcinoma or bladder carcinoma or urinary tract carcinoma or urethra carcinoma” combined with “antihypertensive medications or angiotensin-converting enzyme inhibitors or angiotensin receptor blockers or beta-blockers or calcium-channel blockers or diuretics.” Additionally, a manual search of the references cited in relevant original and review articles was conducted.

### Selection criteria

The eligible studies were required to meet all of the following inclusion criteria: (1) assessing the association between exposure to antihypertensive medications (ACEI, ARB, BB, CCB, diuretics) and urinary bladder neoplasms and kidney cancer (renal cell carcinomas) risk, (2) original case-control study, nested case-control study, and cohort study, (3) reporting the odds ratio (OR) or RR with corresponding 95% confidence intervals (CI). Renal pelvis and ureter cancers were excluded as they were mostly of transitional cell type and had an etiology comparable to bladder cancer than renal cell cancer. When multiple studies included overlapping data, the latest and most complete study was included. Published letters, editorials, abstracts, reviews, case reports, and expert opinions were not included. The discrepancies between two investigators were resolved through discussion or in consultation with a third reviewer.

### Data extraction and quality assessment

For each included study, the following baseline characteristics were extracted and recorded: first author’s name, publication date, study design, source of participants, study period, age, number of participants, class of medication exposure, assessment of outcome, estimated effect size (OR, RR), corresponding 95% CI, and adjustments for confounders. The risk estimates, adjusted by multiple factors, were preferably extracted from each eligible study. Moreover, to investigate the dose-response relationship, we extracted the cumulative duration outcomes observed with any class of antihypertensive medications therapy. To assess the quality of the included studies, NOS was used and NOS score > 6 was regarded as a high-quality study.

### Statistical analysis

We used an RR with 95% CI to estimate the associations between each class of antihypertensive medications and bladder/kidney cancer risk. In order to explore whether gender, smoking, and hypertension affected this association, subgroup analyses were performed.

We used generalized least squares trend regression models to perform dose-response analyses and investigate the trend between the duration of antihypertensive medications therapy and cancer risk [55, 56]. The restricted cubic spline model with 3 knots at 25%, 50% and 75% percentiles of the whole distribution was conducted to explore the potential non-linear dose-response association. The null hypothesis that the coefficient of the second spline was equal to zero was tested to calculate the *P*-value of non-linearity [57, 58]. A pooled risk estimate was calculated for a standardized increment with the duration of antihypertensive medications therapy. This analysis used data from the RR and 95% CI, number of cases, number of overall participants, and median or mean duration of antihypertensive medications therapy (in years) for each group.

Heterogeneity across the eligible studies was assessed using Cochran’s Q test and I^2^ statistic. The criterion of a *P*-value < 0.05 or I^2^ > 50% indicated significant heterogeneity [59, 60]. If significant heterogeneity was detected, a random-effects model was used, otherwise, a fixed-effects model was employed [61]. Publication bias was examined with Begg’s and Egger’s regression tests [62]. Sensitivity analyses were conducted to determine the effect of each study and the stability of the meta-analysis results. Statistical analyses were performed using STATA software (version 12.0; STATA Corp LP, College Station, TX)

## Supplementary Material

Supplementary Figures
