# Supplementary material for: Antihypertensive medications are associated with the risk of kidney and bladder cancer: a systematic review and meta-analysis
Source: Aging (Albany NY). 2020 Jan 22;12(2):1545–62. doi: 10.18632/aging.102699 (PMC7053620; doi:10.18632/aging.102699)
Supplement: Supplementary Figures [file aging-12-102699-s001..pdf]

SUPPLEMENTARY FIGURES

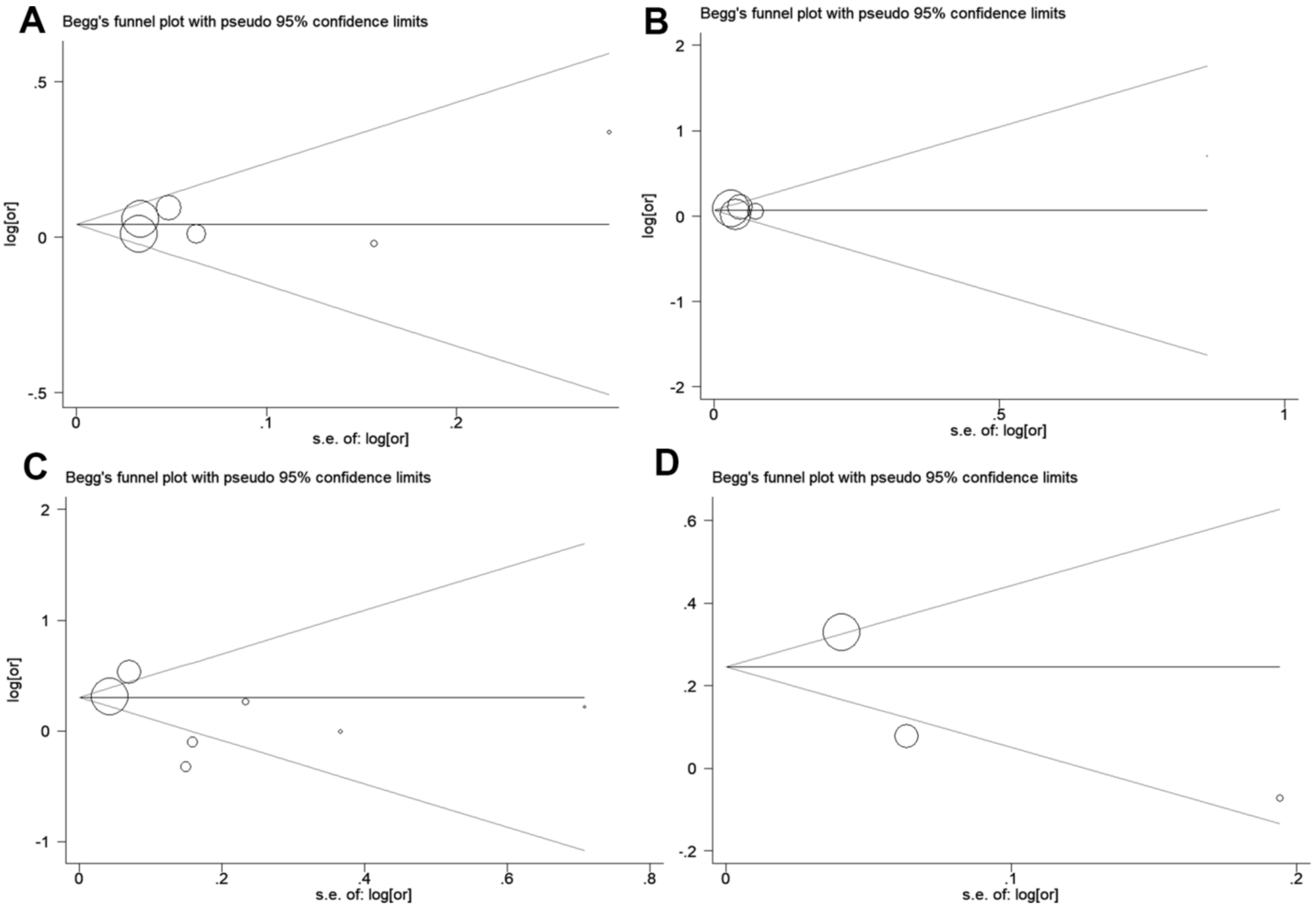

Supplementary Figure 1. Begg's funnel plot of association between using each class of antihypertensive medications and bladder cancer risk: (**A**) ACEI and bladder cancer risk; (**B**) ARB and bladder cancer risk; (**C**) CCB and bladder cancer risk; (**D**) diuretics and bladder cancer risk.

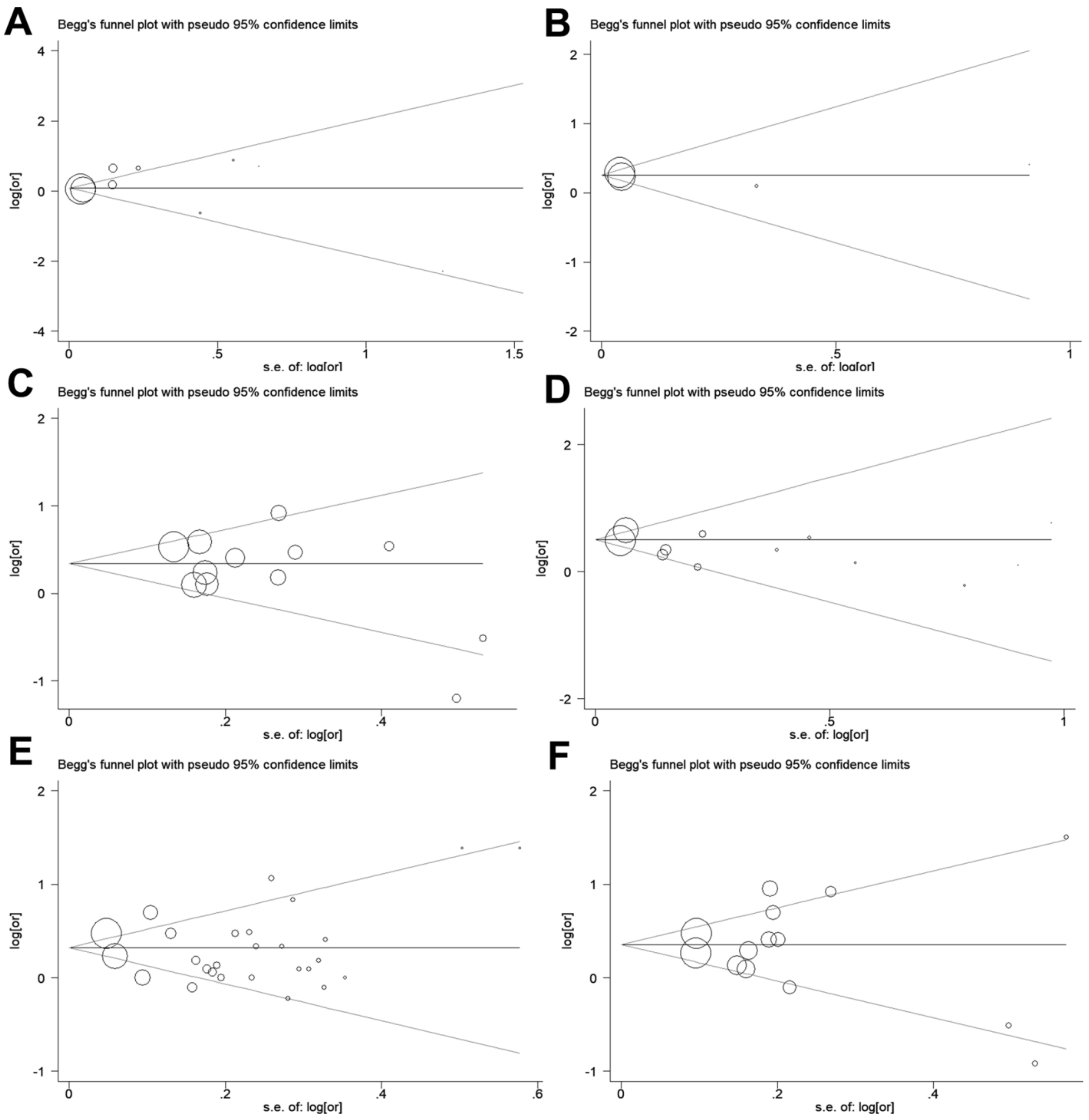

Supplementary Figure 2. Begg's funnel plot of association between using each class of antihypertensive medications and kidney cancer risk: (**A**) ACEI and kidney cancer risk; (**B**) ARB and kidney cancer risk; (**C**) BB and kidney cancer risk; (**D**) CCB and kidney cancer risk; (**E**) diuretics and kidney cancer risk; (**F**) any antihypertensive medications and kidney cancer risk.

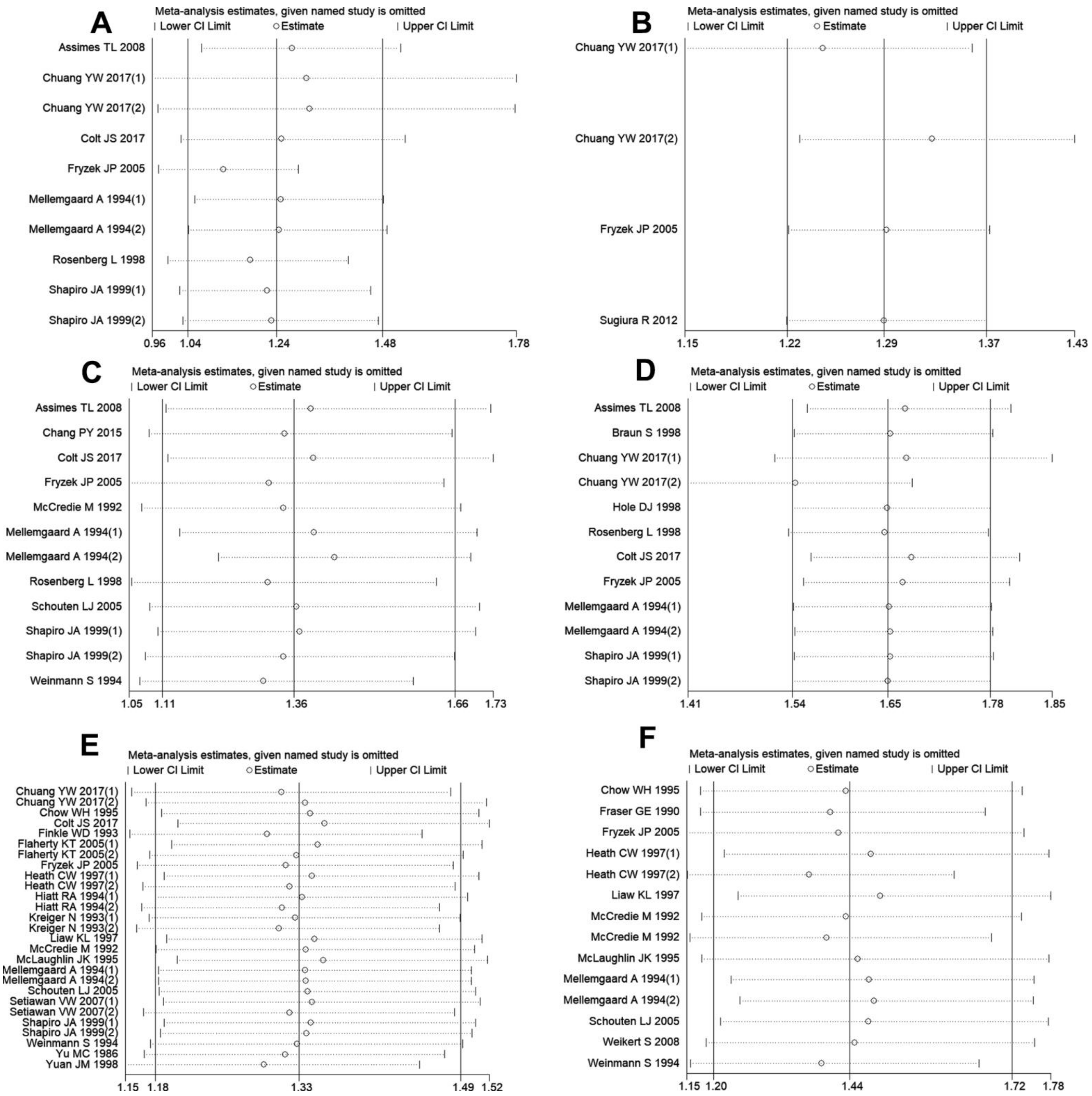

Supplementary Figure 3. Sensitivity analysis of association between using each class of antihypertensive medications and kidney cancer risk: (**A**) ACEI and kidney cancer risk; (**B**) ARB and kidney cancer risk; (**C**) BB and kidney cancer risk; (**D**) CCB and kidney cancer risk; (**E**) diuretics and kidney cancer risk; (**F**) any antihypertensive medications and kidney cancer risk.
